# Blood- and Imaging-Derived Biomarkers for Oncological Outcome Modelling in Oropharyngeal Cancer: Exploring the Low-Hanging Fruit

**DOI:** 10.3390/cancers15072022

**Published:** 2023-03-28

**Authors:** Stefania Volpe, Aurora Gaeta, Francesca Colombo, Mattia Zaffaroni, Federico Mastroleo, Maria Giulia Vincini, Matteo Pepa, Lars Johannes Isaksson, Irene Turturici, Giulia Marvaso, Annamaria Ferrari, Giulio Cammarata, Riccardo Santamaria, Jessica Franzetti, Sara Raimondi, Francesca Botta, Mohssen Ansarin, Sara Gandini, Marta Cremonesi, Roberto Orecchia, Daniela Alterio, Barbara Alicja Jereczek-Fossa

**Affiliations:** 1 Division of Radiation Oncology, IEO European Institute of Oncology IRCCS, Via Ripamonti, 435, 20141 Milan, Italy; 2 Department of Oncology and Hemato-Oncology, University of Milan, 20141 Milan, Italy; 3 Department of Experimental Oncology, IEO European Institute of Oncology IRCCS, 20141 Milan, Italy; 4 Department of Translational Medicine, University of Piemonte Orientale (UPO), 28100 Novara, Italy; 5 Medical Physics Unit, IEO European Institute of Oncology IRCCS, 20141 Milan, Italy; 6 Division of Division of Otolaryngology and Head and Neck Surgery, IEO European Institute of Oncology IRCCS, 20141 Milan, Italy; 7 Radiation Research Unit, IEO European Institute of Oncology IRCCS, 20141 Milan, Italy; 8 Scientific Directorate, IEO European Institute of Oncology IRCCS, 20141 Milan, Italy

**Keywords:** oropharyngeal cancer, lymphocyte-to-monocyte ratio, lymphopenia, radiomics, outcome modeling

## Abstract

**Simple Summary:**

Oropharyngeal squamous cell carcinoma (OPSCC) has one of the most rapidly increasing incidences of any cancer in high-income countries. The aim of this study is to test whether radiomic and blood-derived biomarkers are good candidates for refining the prognostic stratification in OPSCC. The results show that the integration of clinical, immunological, and computed tomography-derived features generally yields an improvement, regardless of the HPV status, in the prognostic stratification of OPSCC patients who are candidates for curative-intent radiotherapy. Specifically, we documented a significant role of the Lymphocyte-to-Monocyte Ratio (LMR) in this population, which has been scarcely investigated in OPSCC, as well as the detrimental effects of lymphopenia and anemia. Results are promising, and model performances compare favorably with available radiomic scores in the same setting. Further investigations on our findings are warranted to validate the results and include a more in-depth study of the prognostic role of the LMR in OPSCC. Future analyses of this dataset are planned to provide a more complete overview of the tumor-immune system interplay.

**Abstract:**

Aims: To assess whether CT-based radiomics and blood-derived biomarkers could improve the prediction of overall survival (OS) and locoregional progression-free survival (LRPFS) in patients with oropharyngeal cancer (OPC) treated with curative-intent RT. Methods: Consecutive OPC patients with primary tumors treated between 2005 and 2021 were included. Analyzed clinical variables included gender, age, smoking history, staging, subsite, HPV status, and blood parameters (baseline hemoglobin levels, neutrophils, monocytes, and platelets, and derived measurements). Radiomic features were extracted from the gross tumor volumes (GTVs) of the primary tumor using pyradiomics. Outcomes of interest were LRPFS and OS. Following feature selection, a radiomic score (RS) was calculated for each patient. Significant variables, along with age and gender, were included in multivariable analysis, and models were retained if statistically significant. The models’ performance was compared by the C-index. Results: One hundred and five patients, predominately male (71%), were included in the analysis. The median age was 59 (IQR: 52–66) years, and stage IVA was the most represented (70%). HPV status was positive in 63 patients, negative in 7, and missing in 35 patients. The median OS follow-up was 6.3 (IQR: 5.5–7.9) years. A statistically significant association between low Hb levels and poorer LRPFS in the HPV-positive subgroup (*p* = 0.038) was identified. The calculation of the RS successfully stratified patients according to both OS (log-rank *p* < 0.0001) and LRPFS (log-rank *p* = 0.0002). The C-index of the clinical and radiomic model resulted in 0.82 [CI: 0.80–0.84] for OS and 0.77 [CI: 0.75–0.79] for LRPFS. Conclusions: Our results show that radiomics could provide clinically significant informative content in this scenario. The best performances were obtained by combining clinical and quantitative imaging variables, thus suggesting the potential of integrative modeling for outcome predictions in this setting of patients.

## 1. Introduction

In recent years, radiation oncology has taken advantage of progress in the fields of biology, computational sciences, and medical engineering [1]. Oropharyngeal squamous cell carcinomas (OPSCCs) can be considered a successful paradigm of the integration of information layers from multiple domains. Specifically, the recognition of the prognostic role of the human papilloma virus (HPV) in the tumor node metastasis (TNM) staging classification has led to the development of de-intensification trials, aiming to reduce unnecessary toxicity in relatively young and long-surviving patients. 

Among novel approaches, immunotherapy (IO) has become a standard of care for recurrent/metastatic head and neck cancers (HNCs), including OPSCC [2,3]. On these bases, ongoing trials are investigating the use of IO in this site, both in its early and advanced stages [4]. However, the estimated percentage of patients achieving a durable response to IO is still low [4,5,6], which suggests the need for additional predictive biomarkers than the already validated PDL-1 [7]. Of these, some blood count parameters (e.g., neutrophil to lymphocyte ratio, monocyte to lymphocyte ratio) have been associated with response to IO in several solid neoplasms, including melanoma and non-small cell lung cancers [8]. While dedicated studies on OPSCC are currently lacking, preliminary evidence suggests that the same association may be valid also in head and neck malignancies, where—as an example—a higher neutrophil-to-lymphocyte ratio (NLR) is associated with poorer outcomes in terms of overall survival (OS) and progression-free survival (PFS) [8]. Other than their potential role as predictors of IO response, blood biomarkers are good candidates for refining the prognostic stratification in OPSCC [9,10,11]. Such information may contribute to identifying patients prone to disease progression who may benefit from the addition of IO or other systemic therapies to optimize healthcare costs and, ultimately, to enhance the therapeutic ratio of clinical indications in this clinical setting. Notably, blood-derived biomarkers are non-invasive, widely available, easily reproducible, and relatively inexpensive. 

Such appealing characteristics are shared with imaging-derived biomarkers, collectively defined as radiomic features, i.e., quantitative parameters that can be extracted from routinely acquired medical images through dedicated software [12,13]. Starting in the early 2010s, an increasing body of evidence has shown the association between radiomic and biological features, including but not limited to necrosis, mitotic rate, and mutational status [12,14,15]. In addition, the association with clinically relevant endpoints (e.g., OS, treatment response) has been extensively investigated in most cancer types, with promising results [16,17,18,19]. While several pitfalls are currently preventing the implementation of radiomics in the clinical workflow [20], ongoing methodological research will arguably allow a progressive transition of radiomics from a hypothesis-generating branch of medical imaging to an actionable clinical tool. Considering OPSCC, a recent systematic review has shown a predominance of studies focusing on computed tomography (CT)-based radiomics [21], with a median number of included patients of 86 (interquartile range, IQR: 41–207) and only seven works focusing on radiation oncology. Therefore, there is a largely unmet need to further explore radiomic applications in homogenous OPSCC cohorts treated with radiotherapy (RT) to understand whether the addition of imaging-derived parameters may contribute to the refinement of outcome prediction. 

As a part of this evolving scenario, this study intends to integrate information from clinical parameters, blood-derived biomarkers, and radiomic features to predict OS and locoregional PFS (LRPFS) in OPSCC treated with curative-intent RT at a single tertiary cancer center. Specific aims are as follows:

- Build prognostic models, including clinical data and blood-derived biomarkers (e.g., NLR), to assess their role in combination with already known patient- and tumor-related parameters.
- Build prognostic models to test the association between computed tomography (CT)-based radiomic features and clinical outcomes of interest (namely, OS and LRPFS).
- Build unified prognostic models integrating clinical data, blood-derived biomarkers, and CT-based radiomic features to explore their association with the above-mentioned clinical outcomes of interest.

## 2. Patients and Methods

### 2.1. Participants and Clinical Outcomes of Interest

This study included patients diagnosed with OPSCC who were treated with curative-intent RT at the Radiation Oncology department of the European Institute of Oncology (IEO) IRCCS between January 2005 and December 2017. To be eligible for the study, patients had to meet the following criteria: (1) age ≥ 18 years; (2) histologically confirmed diagnosis of OPSCC; (3) available CT simulation scans; (4) available clinical and demographical data, tumor characteristics (subsite, clinical staging, histology, grading, HPV status), and pre-RT blood parameters (baseline hemoglobin levels, neutrophils, monocytes, and platelets), and derived measurements; (5) a minimum follow-up of six months; and (6) written informed consent for use of data for clinical research and educational purposes. HPV status was assessed by the detection of p16 on immunohistochemistry (IHC), with a cut-off of at least 70% of cells in a tumor sample. In cases where p16-IHC expression was lower than 70%, HPV-DNA was assessed by polymerase chain reaction (PCR) [22].

Patients who could not have their primary lesion assessed (cTx) and those with secondary or recurrent tumors of the head and neck region were excluded. The administration of either induction chemotherapy or concomitant chemo-RT was allowed. The study was approved by the Institutional Ethical Committee under notification number 94/11.

### 2.2. Computed Tomography Characteristics

All patients underwent a simulation-CT scan with and without administration of intravenous contrast medium, in a supine position, and with a thermoplastic mask to immobilize their heads and shoulders. All CTs were acquired with the GE Healthcare Optima CT580 W scanner and with the same acquisition protocol (120-kV tube voltage, 150-mA tube current, and 2.5-mm slice thickness). 

### 2.3. Tumor Delineation and Feature Extraction

The gross tumor volume segmentations of the OPSCC primaries (GTV-Ts) had been originally performed by a single radiation oncologist with 20 years of experience on HNCs and were independently reviewed for the purpose of this study by a second radiation oncologist with dedicated expertise on HNCs, as well. Notably, macroscopic nodal disease was manually excluded from the volume of interest to overcome possible variabilities from radiomic features heterogeneity between the primary tumor and regional lymph nodes.

Radiomic features were extracted from the non-contrast enhanced CTs using Pyradiomics v3.01 in Python 3.7.10 with the use of Numpy 1.19, SimpleITK 2.0, and PyWavelet 1.1. All features and all image types (i.e., image preprocessing filters) were enabled on all CT scans, as previously reported [23].

### 2.4. Statistical Analysis

Frequencies, medians, and first and third quartiles were used to describe categorical and continuous variables, respectively. Oncological outcomes considered for the analysis were: OS, defined as the time length from diagnosis to death from any cause or last contact at follow-up; LRPFS, defined as the time length from diagnosis to locoregional disease progression or death from any cause or lost contact at follow-up; and Distant Metastasis Free-Survival (DMFS), defined as the time length from diagnosis to distant metastatic disease progression or death. A subgroup analysis was performed for HPV-positive patients, re-classifying cases to the 8th AJCC edition to correctly stage patients.

#### 2.4.1. Feature Selection and Radiomic Score Calculation

Features with near-zero variance and high correlation (Spearman ρ > 0.95) were initially excluded. The remaining features were clustered by an iterative clustering algorithm, grouping features when Spearman ρ > 0.75. In each cluster, only the feature most associated with each of the two investigated outcomes (the lowest *p*-value from the Cox proportional hazard univariate regression model) was retained. The procedure was iterated until the Spearman ρ in finally selected features resulted <0.75. 

A coefficient for each feature was obtained by the Multivariable Cox—Least Absolute Shrinkage and Selection Operator (LASSO) Regression Model, in which a greater coefficient value corresponds to a greater contribution in endpoints. The resulting radiomic score for each patient was calculated by summing the features multiplied by their relative coefficient.

#### 2.4.2. Comparison of Prognostic Models

The study evaluated three prognostic models for each survival endpoint: clinical model—containing only clinical information and blood-derived biomarkers; radiomic model—containing only the radiomic score; clinical-radiomic model—containing both clinical information and a radiomic score.

Univariate Cox proportional hazard regression models were used to test associations between clinical variables and radiomic score, with the endpoints and cut-off points for continuous variables chosen according to clinical significance or, if absent, by using the median value. Variables with *p* ≤ 0.10 in univariate analysis were included in multivariate analysis and retained if the *p*-value was confirmed as ≤0.10. In order to avoid collinearity, only one of the different significantly correlated variables was included in the multivariable model: the one with the lowest *p*-value in the univariate analysis. Risk estimates were quantified by the Hazard Ratio (HR) and 95% confidence intervals (CI). A log-rank test was used to compare survival curves by significant variable strata. For the radiomic score, the median was used to dichotomize the variable into high and low radiomic scores. 

Clinical, radiomic, and clinical-radiomic models were compared by the C-index, a goodness of fit measure for binary outcomes ranging from a very poor predictive model (0.5) to a hypothetically perfect predictive model (1.0). For each clinical-radiomic model, an in-sample 10- and 5-fold cross-validation was implemented for OS, LRPFS, and DMFS, respectively, and repeated 500 times with different random seeds. Since the sample size was small for the subgroup analysis on HPV-positive patients, this latter analysis was only feasible with 3-fold cross-validation. The median and interquartile range (IQR) of the C-Index estimates were reported.

All analyses were considered statistically significant if *p* < 0.05. The statistical analyses were performed using R Software version 4.1.1 (10 August 2021).

## 3. Results

### 3.1. Patients Characteristics

A total of 105 patients met the inclusion criteria and were included in the study. The median age was 59 years (IQR: 52–66). A summary of the patients’ characteristics is provided in Table 1, and a summary of the blood count parameters at baseline is reported in Table 2.

### 3.2. Overall Survival

#### 3.2.1. Whole Population

The median follow-up for OS was 6.3 years (IQR 5.3–7.9). Three radiomic features were included in the radiomic and clinical-radiomic models: original_shape_LeastAxisLength (preprocessing type_feature category_feature name), original_shape_Sphericity, and gradient_glszm_ZoneEntropy. The median radiomic score was 2.26 (IQR 1.99–2.46). 

**Figure 1:**
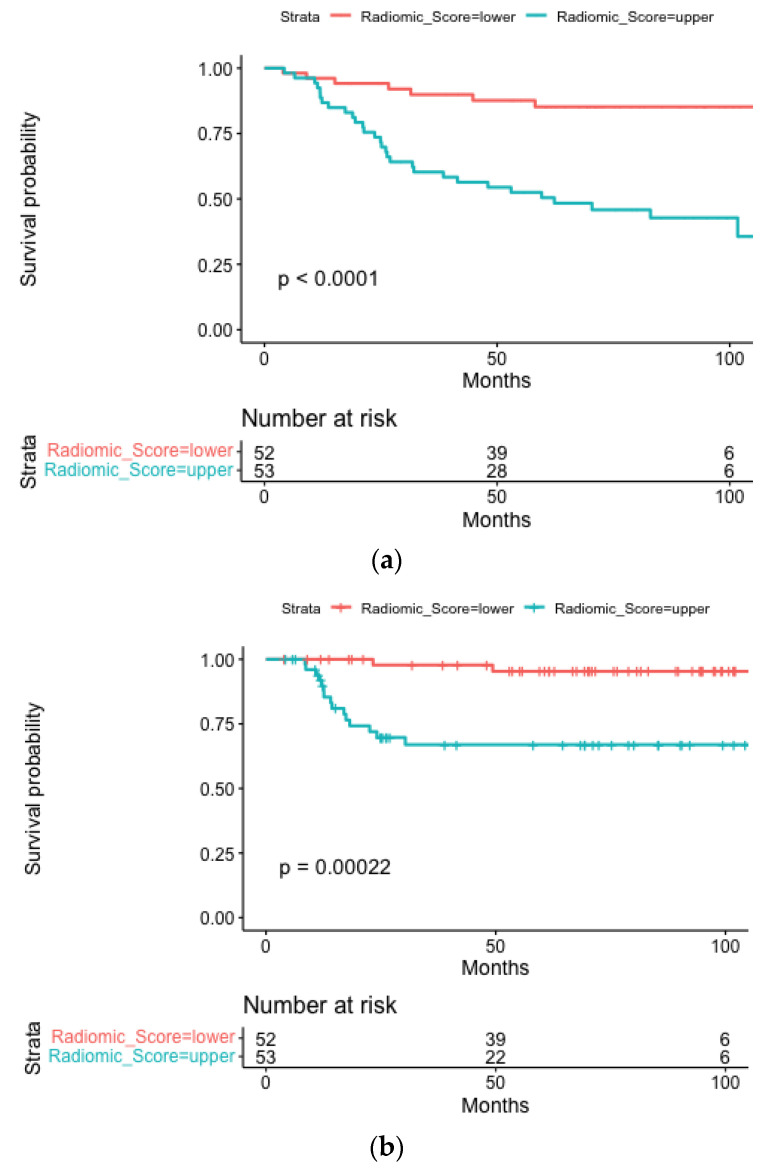

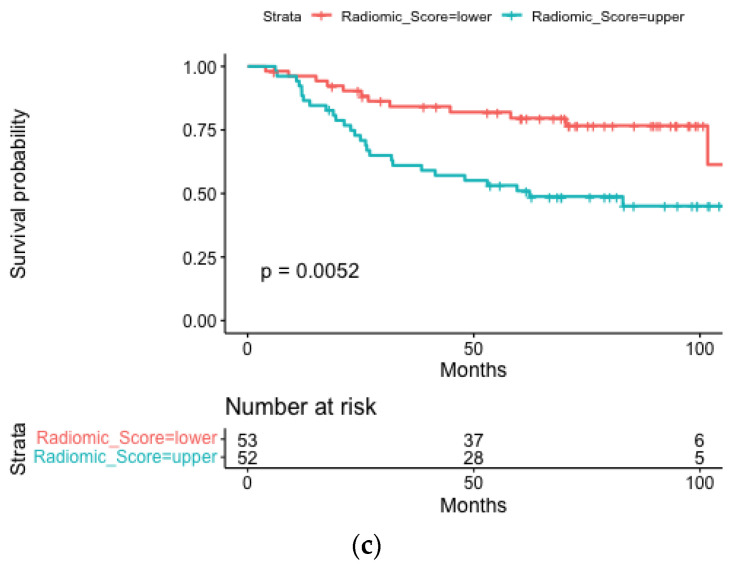
Kaplan-Meier curves stratified according to the radiomics score depicting OS (**a**), LRPFS (**b**) and DMFS (**c**) for the whole population.

A statistically significant difference was observed between a high and low radiomic score (log-rank *p* < 0.0001) for overall survival (Figure 1a). Results from multivariable analysis for OS are presented in Table S1 for the three considered models. While tumor stage IVA (HR = 0.39; 95%CI [0.17–0.94], *p* = 0.04) and positive HPV status (HR = 0.15; 95%CI [0.05–0.42], *p* < 0.0001) were significant positive prognostic factors for OS both in clinical and radiomic models, an increase in radiomic score was found to be negatively associated with OS in both the radiomic and clinical-radiomic models.

Repeated 10-fold cross-validated C-index can be seen in Table 3 for both training and test sets; for OS, the clinical model (median test C-index 0.78, IQR 0.76–0.81) is similar to the pure radiomic model (median test C-index 0.77, IQR 0.75–0.80), while the clinical-radiomic model (median test C-index 0.82, IQR 0.80–0.84) resulted in the best performing one. 

#### 3.2.2. HPV+ Subgroup

**Figure 2:**
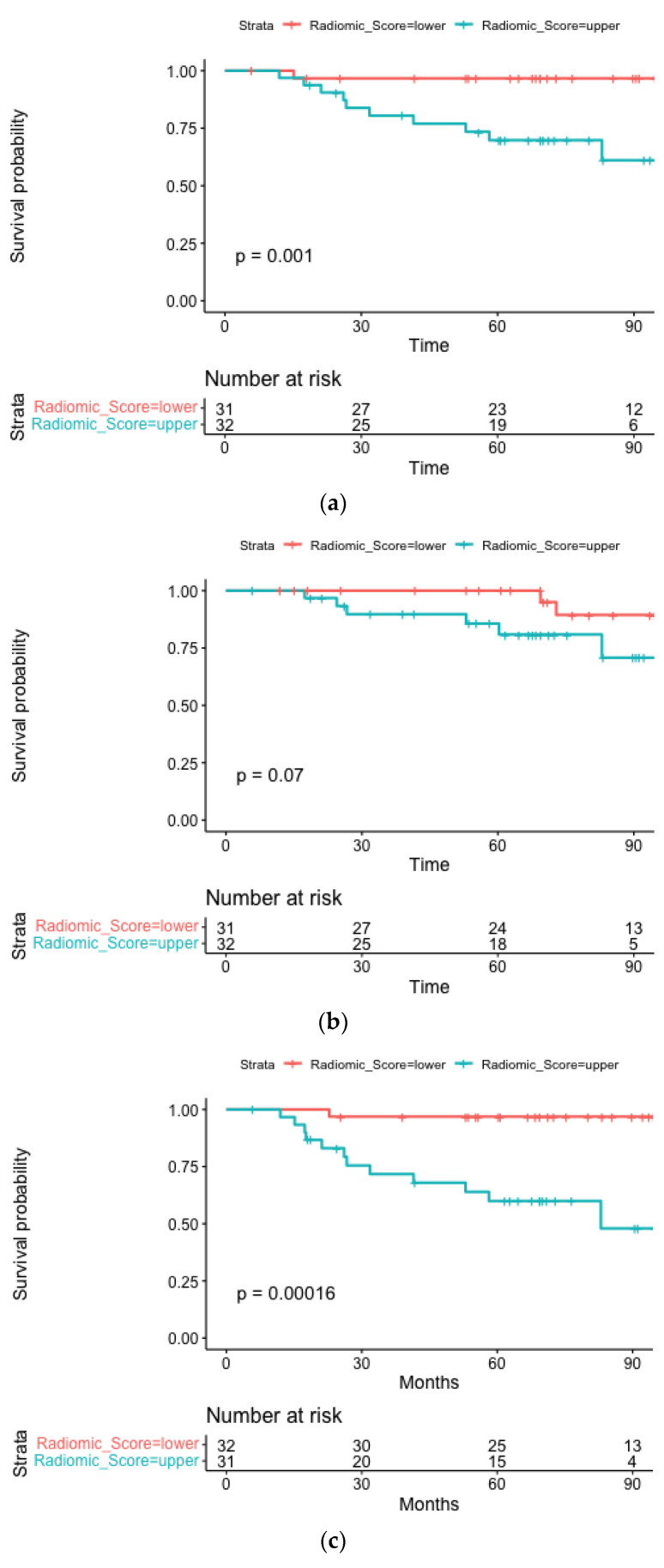
Kaplan-Meier curves stratified according to the radiomics score depicting OS (**a**), LRPFS (**b**), and DMFS (**c**) for the HPV positive subgroup.

The median follow-up time for OS was 6.0 years (IQR 5.1–7.8). Four radiomic features were included in the radiomic and clinical-radiomic models: original_shape_LeastAxisLength log-sigma-1-0-mm-3D_glrlm_LongRunHighGrayLevelEmphasis, wavelet-HLL_gldm_DependenceNonUniformityNormalized, wavelet-HLH_firstorder_Median. The median radiomic score was 4.24 (IQR 3.88–4.64). A statistically significant difference is observed between high and low radiomic scores (log-rank *p* = 0.001) for overall survival (Figure 2a).

The results from multivariable analysis for OS in the HPV+ group (*n* = 63) are presented in Table S2 for the three considered models. Lymphocyte-to-Monocyte Ratio (LMR) values lower than 2.6 resulted in bad prognostic associated variables (HR = 5.60, 95%CI [1.12, 27.9], *p* = 0.04) in the clinical model. In the clinical radiomic model, an increase in radiomic score was significantly negatively associated with OS for HPV+ patients (HR = 4.30, 95%CI [2.02,9.16], *p* < 0.001), while the LMR did not remain significant.

Repeated cross-validated C-index can be seen in Table 4; in contrast to results for the whole cohort, the radiomic model (median test C-index 0.83, IQR 0.80–0.87) outperformed the pure clinical model (median test C-index 0.79, IQR 0.76–0.83), therefore combining clinical and radiomic features (median test C-index 0.86, IQR 0.82–0.89) resulted in the best performing one according to both methods, as in the whole cohort. 

### 3.3. Locoregional Progression-Free Survival

#### 3.3.1. Whole Population

The median follow-up for LRPFS was 5.8 years (IQR, 3.0–7.7). Five radiomic features were included in the radiomic and clinical-radiomic models: original_firstorder_90Percentile, original_firstorder_InterquartileRange, lbp-3D-k_firstorder_10Percentile, wavelet-HLH_firstorder_Median, and wavelet-HHH_firstorder_Median. The median radiomic score was 0.70 (IQR 0.46–0.85). A statistically significant difference is observed between a high and low radiomic score (log-rank *p* = 0.001) (Figure 1b).

The results from multivariable analysis for LRPFS are presented in Table S3 for the three considered models. Tumor stage IVB and age were found to be significantly associated with poor prognosis in disease progression in the clinical model (HR = 4.53; 95% CI [1.47–14], *p* = 0.009 and HR = 1.06; 95% CI [1.01–1.12], *p* = 0.03, respectively); however, these results were not confirmed by adding the radiomic variable. 

A repeated 5-fold cross-validated C-index can be seen in Table 3. The radiomic model (median test C-index 0.80, IQR 0.79–0.82) outperformed both the clinical (median test C-index 0.72, IQR 0.70–0.73) and clinical-radiomic models (median test C-index 0.77, IQR 0.75–0.79).

#### 3.3.2. HPV+ Subgroup

The median follow-up for LRPFS was 5.8 years (IQR 4.5–7.8). Four radiomic features were included in the radiomic and clinical-radiomic models: original_firstorder_InterquartileRange, exponential_firstorder_90Percentile, exponential_firstorder_Entropy, wavelet, and HLL_gldm_DependenceNonUniformityNormalized. The median radiomic score was 2.31 (IQR 2.24–2.42). The radiomic score cut-off was able to divide with borderline statistical significance (log-rank *p* = 0.07) on Kaplan-Meier curves for the two subgroups (Figure 2b).

The results from multivariable analysis for LRPFS in the HPV+ group are presented in Table S4 for the three considered models. Younger age and higher hemoglobin (HB) were significantly associated with a better LRPFS (HR = 1.07, 95%CI [1.0–1.15], *p* = 0.04; HR = 0.64, 95%CI [0.42–0.97], *p* = 0.04) in the clinical model; however, these results were not confirmed by adding the radiomic variables. High radiomic score resulted significantly associated with worst LRPFS both in the univariate clinical-radiomic model (*p* < 0.001) and in the radiomic one (*p* = 0.023). 

Table 4 shows the 3-fold cross-validated C-index. The radiomic model (C-index 0.77), which outperformed the clinical-radiomic model (C-index 0.65) and the clinical model (C-index 0.66).

### 3.4. Distant Metastasis Free Survival

#### 3.4.1. Whole Population

The median follow-up for DMFS was 6.3 years (IQR 5.3–8.1). Two radiomic features were selected for inclusion in the radiomic and clinical-radiomic models, namely: original_shape_Sphericity and gradient_glszm_ZoneEntropy. The median radiomic score was 0.76 (IQR 0.69–0.82); the threshold could operate a statistically significant discrimination between the subgroups (log-rank *p* = 0.0052), as shown in Figure 1c.

The results from multivariable analysis for this endpoint are presented in Table S5 for all models. Hemoglobin levels and the NLR were both significantly associated with DMFS in the clinical model (HR = 0.72; 95% CI [0.59–0.87], *p* = 0.001 and HR = 1.39; 95% CI [1.15–1.67], *p* = 0.001, respectively), with hemoglobin levels retaining statistical significance following the addition of the radiomic variables. 

Repeated 5-fold cross-validated C-indexes are reported in Table 3. The clinical-radiomic model outperformed individual clinical and radiomic models (median test C-index 0.80, IQR 0.78–0.81).

#### 3.4.2. HPV+ Subgroup

The median follow-up for the HPV+ cohort was 6.1 years (IQR 5.2–7.9). The 20 selected radiomic features derive mainly from the higher-order category (12/20), followed by features belonging to the first-order category (7/20), and from the shape category (1/20). The median radiomic score was 14.7 (IQR 14.1–15.2) and showed a statistically significant ability to discriminate between prognostic subgroups (log-rank *p* = 0.00002), as reported in Figure 2c. 

Multivariable analysis showed a significant contribution of the LMR in the clinical model (HR = 0.47; 95% CI [0.24–0.92], *p* = 0.029), which was not retained in the clinical-radiomic model (Table S6). 

Repeated three cross-validated C-indices are provided in Table 4 and show comparable performance between the radiomic and clinical-radiomic models (a median test C-index of 0.96).

## 4. Discussion

Our results show that the integration of clinical, immunological, and CT-derived features generally yields an improvement in the prognostic stratification of OPSCC patients who are candidates for curative-intent RT. Specifically, we demonstrated that staging maintained its expected prognostic significance in the clinical-radiomic model for both OS and LRPFS. Additionally, HPV status and age were significant determinants of OS and LRPFS, respectively. The use of the radiomic score allowed for good-to-excellent discrimination into two prognostic subgroups, which was applicable for all the outcomes of interest, regardless of the HPV status (0.0001 ≤ log-rank *p* ≤ 0.07). 

Considering immunological biomarkers, the clinical model suggested an association between LMR and OS, which reached statistical significance in the HPV-positive subgroup. The prognostic role of LMR has been previously reported for other cancers, including melanoma, breast cancer, and non-small cell lung cancer [24,25,26]. Albeit apparently common, pathophysiological mechanisms remain poorly understood. Generally, the relative decrease in lymphocytes to monocytes may be caused by cancer-induced dysregulations in hematopoiesis [27,28,29,30,31]. In this immunosuppressive scenario, lymphopenia (i.e., lymphocytes < 1000/mcl) plays a well-documented role and has been extensively associated with a poorer prognosis [32]. Other than tumor-induced lymphopenia, a treatment-related decrease in the number of circulating lymphocytes has a detrimental effect, as confirmed by a recent meta-analysis investigating the effects of radiation on head and neck cancers [10]. 

Overall, LMR may be considered a surrogate of these phenomena, and its value in HNC has recently been confirmed in a 2018 meta-analysis by Tham et al. [11]. In this work, 4260 HNCs of predominantly nasopharyngeal origin were analyzed, and the authors identified a significant association between LMR and OS, disease-specific survival, and LRPFS when the LMR was dichotomized into low and high. Of note, a relatively wide range of LMR cut-offs was reported (namely, 2.475–5.300). On the one hand, this reflects a lack of standardization in the choice of a well-defined threshold; on the other, such discrepancies may derive from differences in patients’ subpopulations, in terms of geographical origin and/or tumor subsite. In our series, the threshold for LMR was derived from the median of the population (namely, 2.6), which however falls within the previously reported ranges. Nevertheless, whether the negative prognostic value of LMR in HNCs applies to OPSCC specifically is yet to be confirmed [11,33,34]. To date, few studies have focused on OPSCC alone [35,36]. Of these, the retrospective work by Tsai et al. endorsed the hypothesis that LMR may act as an independent prognosticator in a cohort of 142 HPV-negative patients with locally-advanced disease, with values below 4.45 being correlated with decreased OS and LRPFS at 5-years [35]. 

The same consideration applies to the absolute lymphocyte count, whose role has been extensively investigated in various cancers, including HNCs [32,37]. In our series, lymphocyte levels below 1000/mcl were associated with borderline significance with a lower LRPFS in the HPV-positive subgroup (*p* = 0.084, results not shown). Consistently, a recent publication by Kreinbrink et al. [9] has applied a pre-treatment lymphocyte increase of 1000 to a population of 158 HPV-positive patients, mostly treated with concomitant chemo-RT. After a median follow-up of 40 months, higher lymphocyte levels significantly correlated with improved OS and LRPFS (*p* = 0.040 and *p* = 0.007, respectively). 

Considering other immunological biomarkers, we identified a statistically significant association between low Hb levels and poorer DMFS in the whole population (*p* = 0.001) and with lower LRPFS in the HPV-positive subgroup (*p* = 0.038). This finding is supported by previous studies on comparable patient populations [38,39,40]. Overall, the physiopathology of this phenomenon remains unclear, and the literature has led to contradictory results [40,41]. 

The NLR was associated with DMFS, regardless of the HPV status in our series, but—quite surprisingly—did not retain any prognostic role for the prediction of either OS or LRPFS. This result is partially contradictory to the findings of comparable series [42,43,44] and may be due to either a peculiar variable distribution in the present dataset and/or to publication bias in the available literature. In addition, the Systemic Immune Inflammation Index (SII) did not show any prognostic role in our series. In this regard, a recent meta-analysis confirms its correlation with oncological outcomes in HNCs, with higher pretreatment values being associated with worse OS, DFS, and PFS [45]. However, the same concerns about the applicability of the OPSCC described above apply to the SII as well, as none of the included studies considered this HN subsite. 

In our work, prognostic information from immunological biomarkers was complemented with that from quantitative image features. In details, the combination of clinical and imaging parameters resulted in consistently improved performance of the clinical-radiomic model for all the outcomes of interest and in both patient populations (namely, the whole group and the HPV-positive subgroup). Notably, the radiomic score alone achieved excellent prognostic stratification in all cases, which suggests that radiomic features could contribute to patients’ stratification and arguably become a part of tailored treatment strategies. 

Among the LASSO-selected features, the majority (16/36) belonged to the higher-order statistics category. According to the terminology proposed by the Image Biomarker Standardization Initiative (IBSI) [46], this is a generic term that includes features of higher mathematical complexity that quantify gray-level zones, runs, and dependencies (GLSZM, GLRLM, and GLDM, respectively). Interestingly, selected higher-order features were associated with OS and DMFS independently of the HPV status and can therefore be considered worthy of further investigation. Almost equally represented (15/36) were features belonging to the first-order statistics class, defined as the “distribution of voxel intensities within the image region” [46]. Specifically, the median was a first-order feature often selected as a prognosticator in our study and expresses the median gray level intensity within the considered ROI (i.e., the GTV). Finally, five of the LASSO-selected features are classified within the shape category, which provides a quantitative description of the 2- and 3-dimensional size and shape of the ROI [46]. Our results show that the LeastAxisLength (namely, the smallest axis length of the ROI-enclosing ellipsoid) was associated with OS regardless of the HPV status, thus suggesting its potential prognostic value in OPSCC.

Our results also show that clinically relevant features were more often selected from preprocessed images than from the original image. This is coherent with the use of image filtering as a strategy to enhance image properties and to unveil otherwise undetectable information, as previously observed by our group [23]. Specifically, different permutations of the wavelet filter seem to yield the highest informative content as compared with other modalities (e.g., gradient, lbp-3D, and log-sigma). Admittedly, this strategy has been quite unexplored in HNC radiomics. We believe its application to wider datasets may contribute to the full exploitation of radiomic potentials in this clinical setting and help lay the foundation for a more solid methodological basis, as it is being realized in the context of other cancer types, such as the lung [23,47,48]. 

Despite being innovative in nature, this work is not without limitations. Firstly, this is a retrospective study, and at the time of the analysis, it was not possible to retrieve missing information on the HPV status. Another bias may derive from the relatively high proportion of HPV-positive patients. If this mirrors the increasing burden of HPV-related OPSCC in Western countries [49], concerns can be raised regarding the goodness of fit of the proposed models in HPV-negative populations. In this regard, a sub-analysis on the HPV-negative patients in our cohort was not possible due to the low number of patients in this group. Finally, there is a current lack of validation on external datasets, which would help to achieve higher robustness. 

On the other hand, several strengths can be acknowledged, especially considering its novelty. Indeed, to the best of our knowledge, this is the first attempt to integrate blood-derived immunological biomarkers, clinical parameters, and radiomics. Results are promising, and model performances compare favorably with available radiomic scores in the setting of HNC [50,51,52]. Moreover, the sample size is larger than the average of radiomic-based studies focusing on OPSCC [21], and all CTs were acquired with the same scanner and following a standardized acquisition protocol. As a further quality assurance measurement, all segmentations were performed by a single experienced radiation oncologist and reviewed by a second radiation oncologist with dedicated expertise in HNCs; therefore, no significant inter-observer bias applies. 

Overall, further investigations on our findings are warranted and include a more in-depth study of the prognostic role of the LMR in OPSCC and the validation of methodologically sound radiomic pipelines for this clinical setting (e.g., determination of the impact of image filtering, analysis of the volume-confounding effect). Future analyses of this dataset are planned to provide a more complete overview of the tumor-immune system interplay. Not only tumor-infiltrating lymphocytes from the intra- and peri-tumoral spaces will be retrieved from biopsy and surgical specimens, but radiomic features will be extracted from GTV expansions to match imaging and biological information and explore possible associations between these parameters and their prognostic value. 

## 5. Conclusions

Our study clearly indicates that the integration of blood-derived parameters and radiomic features results in a refined prediction of oncological outcomes in OPSCC, regardless of the HPV status. Specifically, we documented a significant role of the LMR in this population, which has been scarcely investigated in OPSCC, as well as the detrimental effects of lymphopenia and anemia. Our findings show that the first-order statistics category (i.e., the distribution of voxel intensities within a volume of interest) may be associated with oncological outcomes. In addition, the use of filtered CT images (i.e., preprocessing) seems to provide additional informative content. These methodological notes require dedicated investigations; external validation of the results is strongly encouraged, as well. 

## Figures and Tables

**Table 1:** Patient cohort characteristics.

Patients Characteristics		
		*n* (%)
**Gender**		
	Male	74 (70.5)
	Female	31 (29.5)
**Tumor subsite**		
	Tonsil	54 (51.4)
	Base of the tongue	38 (36.2)
	Glosso-epiglottic vallecula	1 (0.9)
	Soft palate	7 (6.7)
	Palatine pillar	2 (1.9)
	Lateral wall	3 (2.9)
**Smoking habits**		
	Never-smoker	29 (27.6)
	Smokers	29 (27.6)
	Former-smokers	27 (25.7)
	NA	20 (18.9)
**Grading**		
	1	2 (1.9)
	2	15 (14.3)
	3	46 (43.8)
	NA	42 (40.0)
**Clinical T**		
	cT1	25 (23.8)
	cT2	40 (38.1)
	cT3	10 (9.5)
	cT4	27 (25.7)
	NA	3 (2.9)
**Clinical N**		
	cN0	8 (7.6)
	cN1	14 (13.4)
	cN2	65 (61.9)
	cN3	10 (9.5)
	NA	8 (7.6)
**Stage (7th ed, 2010)**		
	I	2 (1.9)
	II	4 (3.8)
	III	9 (8.6)
	III/IVa	3 (2.9)
	IVa	73 (69.5)
	IVb	14 (13.3)
**HPV status**		
	HPV+	63 (60.0)
	HPV−	7 (6.7)
	NA	35 (33.3)
**Stage (7th ed, 2010) of HPV+ patients**		
	I	32 (50.8)
	II	11 (17.5)
	III	20 (31.7)
**Site of recurrence**		
	Local recurrence	5 (0.05)
	Regional recurrence	3 (0.03)
	Locoregional recurrence	9 (0.09)
	Distant progression	14 (0.13)
**Chemotherapy**		
	Induction + concomitant	13 (12.4)
	Concomitant	79 (75.2)
	None	13 (12.4)
		**Median (IQR)**
**BMI**		
	Baseline	26.37 (23.74–29.41)
	End of RT	24.03 (21.80–26.20)
**Weight (Kg)**		
	Baseline	79 (67–85)
	End of RT	73 (60–79)

List of Abbreviations: BMI: Body mass index; HPV: Human papillomavirus; IQR: Interquartile range.

**Table 2:** Summary of blood values for the whole cohort.

	Median	IQR
Hemoglobin (g/dL)	13.85	12.53–15.20
Neutrophil (cells/μL)	4560	3660–6030
Lymphocites (cells/μL)	1640	1195–1995
Monocites (cells/μL)	580	445–760
Platelets (10^3^ cells/μL)	233	199–297.5
Neutrophil/Lymphocites	2.98	2.12–4.00
Lymphocites/Monocites	2.64	2.13–3.56
Platelets/Lymphocites	153.85	123.73–190.41

List of abbreviations: IQR: Interquartile range.

**Table 3:** Summary of the model performances for the whole population.

OS	10-Fold Cross-Validation				
	Repeated 500 Times				
	C-Index Train	C-Index Test	Median C-Index Test	IQR C-Index Test	
**Radiomic model**	0.75	0.74	0.77	0.75	0.8
**Clinical model**	0.8	0.77	0.78	0.76	0.81
**Clinical radiomic model**	0.84	0.79	0.82	0.8	0.84
**LRPFS**	**5-Fold Cross-Validation**				
	**C-Index Train**	**C-Index Test**	**Median C-Index Test**	**IQR C-Index Test**	
** Radiomic model **	0.77	0.76	0.8	0.79	0.82
** Clinical model **	0.75	0.66	0.72	0.7	0.73
** Clinical Radiomic model **	0.81	0.66	0.77	0.75	0.79
** DMFS **	**5-Fold Cross-Validation**				
	**C-Index Train**	**C-Index Test**	**Median C-Index Test**	**IQR C-Index Test**	
**Radiomic model**	0.73	0.72	0.72	0.72	0.72
**Clinical model**	0.77	0.68	0.75	0.73	0.77
**Clinical radiomic model**	0.81	0.82	0.80	0.78	0.81

List of abbreviations: BMI: Body Mass Index; CI: Confidence interval; DMFS: Distant Metastasis Free-Survival HPV: Human Papillomavirus; IQR: Interquartile Range; L: Lower; LRPFS: Locoregional Progression-Free Survival; OS: Overall Survival.

**Table 4:** Summary of the model performances for the HPV positive subgroup.

	3-Folds Cross-Validation				
			Repeated 500 Times		
HPV+ OS	C-Index Train	C-Index Test	Median C-Index Test	IQR C-Index Test	
** Radiomic model **	0.8	0.84	0.83	0.8	0.87
** Clinical model **	0.79	0.76	0.79	0.76	0.83
** Clinical Radiomic model **	0.9	0.88	0.86	0.82	0.89
**LRPFS**	**C-Index Train**	**C-Index Test**	Median C-index test *	IQR C-index test *	
**Radiomic Model**	0.74	0.77			
**Clinical Model**	0.91	0.66			
**Clinical Radiomic Model**	0.91	0.65			
**DMFS**	**C-Index Train**	**C-Index Test**	**Median C-index test**	**IQR C-index test**	
**Radiomic Model**	0.95	0.94	0.96	0.94	0.97
**Clinical Model**	0.68	0.66	0.70	0.67	0.73
**Clinical Radiomic Model**	0.95	0.94	0.96	0.94	0.97

List of abbreviations: BMI: Body Mass Index; CI: Confidence Interval; DMFS: Distant Metastasis Free-Survival; HPV: Human Papillomavirus; IQR: Interquartile Range; L: Lower; LRPFS: Locoregional Progression-Free Survival; OS: Overall Survival. * The number of events was not sufficient to perform repeated cross validation.

## Data Availability

The data presented in this study is available on request from the corresponding author.

## Supplementary Materials

The following supporting information can be downloaded at: https://www.mdpi.com/article/10.3390/cancers15072022/s1, Table S1: Results from multivariate analysis for overall survival in the whole cohort; Table S2: Results from multivariate analysis for overall survival in the HPV positive subgroup; Table S3: Results from multivariate analysis for locoregional progression-free survival in the whole cohort; Table S4: Results from multivariate analysis for locoregional progression-free survival in the HPV positive subgroup; Table S5: Results from multivariate analysis for distant metastasis-free survival in the whole population; Table S6: Results from multivariate analysis for distant metastasis-free survival in the HPV positive subgroup.

## Abbreviations

**CI**	Confidence Interval
**CT**	Computed Tomography
**CV**	Cross Validation
**DMFS**	Distant Metastasis Free-Survival
**GTV**	Gross Tumor Volume
**HB**	Hemoglobin
**HNCs**	Head and Neck Cancers
**HPV**	Human Papilloma Virus
**HR**	Hazard Ratio
**IBSI**	Biomarker Standardization Initiative
**IO**	Immunotherapy
**IQR**	Interquartile Range
**LASSO**	Least Absolute Shrinkage and Selection Operator
**LMR**	Lymphocyte-to-Monocyte Ratio
**LRT**	Likelihood Ratio Test
**LRPFS**	Locoregional Progression-Free Survival
**NLR**	Neutrophil-to-Lymphocyte Ratio
**OPSCC**	Oropharyngeal Squamous Cell Cancer
**OS**	Overall Survival
**PFS**	Progression Free Survival
**PLR**	Platelet-to-Lymphocyte Ratio
**PLT**	Platelets
**ROI**	Region of Interest
**RT**	Radiotherapy
**SII**	Systemic Immune Inflammatory Index
**TNM**	Tumor Node Metastasis
