# Supplementary material for: Blood- and Imaging-Derived Biomarkers for Oncological Outcome Modelling in Oropharyngeal Cancer: Exploring the Low-Hanging Fruit
Source: Cancers (Basel). 2023 Mar 28;15(7):2022. doi: 10.3390/cancers15072022 (PMC10093133; doi:10.3390/cancers15072022)
Supplement: Supplementary file 1 [file cancers-15-02022-s001.zip › cancers-2237079-supplementary.pdf]

Table S1. Results from multivariate analysis for Overall Survival in the whole cohort.

	Clinical Model					Clinical-Radiomic model					Radiomic model				
	**N**	**Event N**	**HR[1]**	**95% CI[1]**	**p-value**	**N**	**Event N**	**HR[1]**	**95% CI[1]**	**p-value**	**N**	**Event N**	**HR[1]**	**95% CI[1]**	**p-value**
**Sex**															
Women	31	5	—	—		31	5	—	—						
Men	74	34	2.57	0.95, 6.92	0.062	74	34	1.8	0.65, 5.00	0.262					
**Age**	105	39	1.03	1.00, 1.07	0.076	105	39	1.03	0.99, 1.07	0.118					
**Tumour stage**															
I-II-III	18	9	—	—		18	9	—	—						
IVA	73	20	0.39	0.17, 0.94	**0.035**	73	20	0.28	0.11, 0.69	**0.006**					
IVB	14	10	1.32	0.46, 3.78	0.61	14	10	1.25	0.42, 3.71	0.693					
**HPV Status**															
Negative	7	6	—	—		7	6	—	—						
Positive	63	12	0.15	0.05, 0.42	**<.001**	63	12	0.21	0.07, 0.61	**0.004**					
**Lymphocyte/Monocyte Ratio**															
≥2.6	52	12	—	—		52	12	—	—						
<2.6	43	21	2.17	0.98, 4.79	0.056	43	21	1.64	0.71, 3.81	0.246					
**Radiomic Score**						105	39	9.62	3.27, 28.3	**<.001**	105	39	15.5	6.00, 39.9	**<.001**

[1] HR = Hazard Ratio, CI = Confidence Interval

n = 105; N events = 39.

Table S2. Results from multivariate analysis for Overall Survival on the HPV positive subgroup.

	Clinical model					Clinical Radiomic model					Radiomic model				
	**N**	**Event N**	**HR[1]**	**95% CI[1]**	**p-value**	**N**	**Event N**	**HR[1]**	**95% CI[1]**	**p-value**	**N**	**Event N**	**HR[1]**	**95% CI[1]**	**p-value**
**Age**	63	12	1.05	0.99, 1.12	0.102	63	12	1.02	0.95, 1.10	0.502					
**Lymphocyte/Monocyte Ratio**															
**≥2.6**	34	2	—	—		34	2	—	—						
**<2.6**	27	9	5.60	1.12, 27.9	**0.036**	27	9	3.49	0.61, 19.9	0.160					
**Radiomic Score**						63	12	4.30	2.02, 9.16	**<.001**	63	12	5.71	2.80, 11.6	**<.001**

[1]HR = Hazard Ratio, CI = Confidence Interval

n = 63.0; N events = 12.0.

Table S3. Results from multivariate analysis for Locoregional Progression Free Survival in the whole cohort.

	Clinical model					Clinical Radiomic model					Radiomic model				
	**N**	**Event N**	**HR1**	**95% CI1**	**p-value**	**N**	**Event N**	**HR1**	**95% CI1**	**p-value**	**N**	**Event N**	**HR1**	**95% CI1**	**p-value**
**Age**	105	17	1.06	1.01, 1.12	**0.032**	105	17	1.05	0.99, 1.11	0.106					
**Stage (VII)**															
IVA	73	8	—	—		73	8	—	—						
IVB	14	5	4.53	1.47, 14.0	**0.009**	14	5	3.01	0.87, 10.4	0.081					
I-II-III	18	4	2.15	0.64, 7.19	0.215	18	4	1.54	0.44, 5.47	0.500					
**Radiomic_Score**						105	17	6.45	2.22, 18.7	**0.001**	105	17	9.79	3.62, 26.5	**<.001**

[1]HR = Hazard Ratio, CI = Confidence Interval

n = 105; N events = 17.0.

Table S4. Results from multivariate analysis for Locoregional Progression Free Survival in HPV positive subgroup.

	Clinical model					Clinical Radiomic model					Radiomic model				
	**N**	**Event N**	**HR[1]**	**95% CI[1]**	**p-value**	**N**	**Event N**	**HR[1]**	**95% CI[1]**	**p-value**	**N**	**Event N**	**HR[1]**	**95% CI[1]**	**p-value**
Age	61	8	1.07	1.00, 1.15	**0.040**	61	8	1.12	1.03, 1.21	**0.009**					
Lymphocytes < 1000	61	8	0.99	0.98, 1.00	0.084	61	8	0.99	0.98, 1.01	0.383					
**Hb**	61	8	0.64	0.42, 0.97	**0.038**	61	8	0.86	0.53, 1.39	0.533					
**Radiomic_Score**						61	8	11.1	1.39, 89.2	**0.023**	63	8	11.5	2.98, 44.4	**<.001**

n = 61.0; N events = 8.00; statistic.log = 15.1; p.value.log = 0.002; statistic.sc = 18.5; p.value.sc = 0.000; statistic.wald = 15.0; p.value.wald = 0.002; statistic.robust = NA; p.value.robust = NA; R^2 = 0.219; r.squared.max = 0.616; c-index = 0.900; c-index SE = 0.038; Log-likelihood = -21.7; AIC = 49.3; BIC = 49.6; No. Obs. = 61

[1] HR = Hazard Ratio, CI = Confidence Interval.

Table S5. Results from multivariate analysis for Distant Metastasis Free Survival in the whole population.

	Clinical model					Clinical Radiomic model					Radiomic model				
	**N**	**Event N**	**HR[1]**	**95% CI[1]**	**p-value**	**N**	**Event N**	**HR[1]**	**95% CI[1]**	**p-value**	**N**	**Event N**	**HR[1]**	**95% CI[1]**	**p-value**
**Hb**	95	35	0.72	0.59, 0.87	**0.001**	95	35	0.75	0.62, 0.90	**0.002**					
**Gender**															
Female	29	3	-	-		29	3	-	-						
Male	66	32	6.52	1.97, 21.5	**0.002**	66	32	4.58	1.34, 15.6	**0.015**					
**Neutr_Lymph**	95	35	1.39	1.15, 1.67	**0.001**	95	35	1.32	1.07, 1.61	**0.008**					
**Radiomic_Score***						95	35	1.82	1.25, 2.66	**0.002**	105	41	2.34	1.68, 3.27	**0.000**

n = 95.0; N events = 35.0; statistic.log = 32.4; p.value.log = 0.000; statistic.sc = 31.9; p.value.sc = 0.000; statistic.wald = 27.3; p.value.wald = 0.000; statistic.robust = NA; p.value.robust = NA; R^2 = 0.289; r.squared.max = 0.949; c-index = 0.755; c-index SE = 0.038; Log-likelihood = -125; AIC = 257; BIC = 261; No. Obs. = 95

*HR by 0.1 increase of Radiomic Score.

Table S6. Results from multivariate analysis for Distant Metastasis Free Survival in the HPV positive subgroup.

	Clinical model					Clinical Radiomic model					Radiomic model				
	N	Event N	HR[1]	95% CI[1]	p-value	N	Event N	HR[1]	95% CI[1]	p-value	N	Event N	HR[1]	95% CI[1]	p-value
**Lymp_Mono**	61	13	0.47	0.24, 0.92	**0.029**	61	13	0.61	0.26, 1.45	0.262					
**Radiomic_Score***						61	13	1.35	1.18, 1.55	**0.000**	63	14	1.38	1.21, 1.59	**0.000**

n = 61.0; N events = 13.0; statistic.log = 6.11; p.value.log = 0.013; statistic.sc = 5.00; p.value.sc = 0.025; statistic.wald = 4.78; p.value.wald = 0.029; statistic.robust = NA; p.value.robust = NA; R^2 = 0.095; r.squared.max = 0.794; c-index = 0.697; c-index SE = 0.064; Log-likelihood = -45.1; AIC = 92.1; BIC = 92.7; No. Obs. = 61

*HR by 0.1 increase of Radiomic Score.
